# The role of social-psychological factors of victimity on victimization of online fraud in China

**DOI:** 10.3389/fpsyg.2022.1030670

**Published:** 2022-12-23

**Authors:** Zhi Zhang, Zhi Ye

**Affiliations:** ^1^Department of Investigation, Zhejiang Police College, Hangzhou, China; ^2^New Crime Research Center, Zhejiang Police College, Hangzhou, China; ^3^School of Marxism, Zhejiang Police College, Hangzhou, China

**Keywords:** online fraud, victimity, victimization, China, psychology

## Abstract

**Introduction:**

The high incidence of online fraud in China poses a great threat to the social stability and property security of society. Victimity is the state or mindset of victims, referring to the various conditions related to being victims of crimes. Examining the association between the inherent and extrinsic factors of victimity and victimization of online fraud is of great importance for revealing the psychological mechanism of victimization and drawing up preventative measures.

**Methods:**

Through the questionnaire survey of 504 online fraud victims and 523 non-victims, univariate analysis and logistic regression analysis were used to investigate the risk factors correlated with victimization.

**Results:**

Age, education, and social support are positively correlated with fraud victimization, while impulsiveness, trust tendency, smartphone usage, and negative life experiences are negatively correlated with fraud victimization. Subsequent regression analyses showed that all the factors above, except for smartphone usage, are significant predictors for online fraud victimization.

**Discussion:**

Our conceptual model and empirical results demonstrate the important role of victimity in making an individual become a victim and help clarify the mixed findings of previous studies on the risk factors for online fraud.

## 1 Introduction

Recently, internet technology has developed and spread rapidly in China. As of December 2021, the number of internet users in China reached 1.032 billion, and the internet penetration rate reached 73.0% (China Internet Network Information Center, 2022). Types of social media shed light on different victimization methods (Lee, 2021a). Traditional crimes are transferring rapidly to non-contact crimes through the medium of telecommunications and the internet, and new crimes such as online fraud, gambling, and violence are increasing. Among them, online fraud has become the main type of crime that endangers the safety of people’s lives and property and disrupts the network order (Fan and Yu, 2021). The Chinese government attaches great importance to cracking down on new illegal and criminal activities in telecommunications and the internet (General Office of Central Committee of the Communist Party of China [CCCPC], 2022). How to effectively control online fraud has become a real problem that law enforcement departments at all levels must think deeply about and focus on solving. However, there are just a few empirical studies on the social-psychological factors contributing to online fraud victimization in the Chinese population (Fan and Yu, 2021; Lee, 2021a,b), which makes it difficult to design effective preventive policy and legal programs. Therefore, the current study explores the key factors and psychological mechanisms of online fraud in China to help the government develop intervention programs to reduce property damage.

Because of the interaction between the criminal and victim in the process of online fraud, the victimity of the victim should be considered a risk factor in the crime. The concept of victimity was first put forward by Mendelssohn, who defined it as the state, quality, or fact of being a victim (Mendelsohn, 1963). Victimity is determined by the victim’s inherent factors and extrinsic factors (Schneider, 1982). The former refers to the victim’s demographic characteristics, psychological characteristics, lifestyle, and other characteristics inherent in individuals. The latter includes the victim’s bad family environment, unhealthy cultural environment of the community, social factors that promote repeat victimization and other objective factors that easily lead to victimization (Li, 2010). The current study attempts to examine the role of inherent factors and extrinsic factors of victimity on online fraud victimization through the comparison of victims and non-victims of online fraud to profile people with high victimity and thus a high likelihood of victimization.

From the perspective of the inherent features of victimity, demographic characteristics are one of the risk factors for online fraud victimization and have been examined by many studies with mixed and inconclusive findings (Ross et al., 2014; Judges et al., 2017; Yin, 2017; Kadoya et al., 2021). For example, Fan and Yu (2021) found that older age, lower education, and being a migrant were associated with a higher risk of fraud victimization. While in Whitty’s (2020) study, age was not a predictive variable for cyberscam victimization and education was a positive predictor, which was also found in Parti’s (2022) study that higher education was not a protective but rather a predictive factor for online fraud in younger respondents. Besides, the findings of Reyns and Randa (2019) showed a negative association between age and fraud victimization. For gender, some studies have suggested that victims of financial fraud are mostly males (Button et al., 2014; Deliema et al., 2019; Whitty, 2020), while others have found no significant effects for gender (Yang, 2017; Fan and Yu, 2021). The inconsistency of the existing conclusions may be related to the differences in the study subjects and types of fraud. As a country with a large population and high incidence of online fraud crime, its sample study on the risk factors of online fraud victimization is representative.

Psychological characteristics are another risk factor contributing to the victimity of fraud victims, such as self-control, impulsiveness, trust tendency, and risk-taking. It is consistently suggested that low self-control, high impulsiveness, and high risk-taking are positively related to victimization (Bossler and Holt, 2010; Ngo and Paternoster, 2011; Pattinson et al., 2011; Alseadoon et al., 2013; Williams et al., 2017; Norris et al., 2019; Mikkola et al., 2021; Parti, 2022). Individuals with low self-control are more likely to engage in online activities and may expose more personal information (Mesch and Dodel, 2018). Impulsivity is a sign of low self-control and impulsive individuals enjoy immediate benefits regardless of the long-term harmful consequences of their actions and are insensitive to the intentions of others (Pratt et al., 2014). For trust tendency it is commonly believed that more trusting people would be more likely to be fraud victims, while the results of trust tendency are surprisingly more mixed. For example, Ross et al. (2014) suggested that increased trust was one of the key areas where older people were more likely to be disproportionately exploited by fraudsters. Other studies consider that trust has a negative or no effect on fraud victimization (Carter and Weber, 2010; Judges et al., 2017). Therefore, the role of psychological characteristics such as impulsivity and trust tendency on the online fraud victimization needs to be examined.

Regarding lifestyle, studies have found that lifestyles such as high internet usage, high online consumer behavior, habitual email response, and having provided money to scammers before predict fraud victimization (Holtfreter et al., 2008; Reisig and Holtfreter, 2013; Vishwanath, 2015; Balleisen, 2018; Parti, 2022). According to routine activities theory, some routine online activities are positive predictors of cyber victimization by exposing potential victims to cybercriminals (Van Wilsem, 2013; Mesch and Dodel, 2018). The integrated lifestyles and routine activities theory (L-RAT) further suggests that the differences in both daily activities and risk taking behavior make some people suitable targets for victimization (Tapp, 2018). However, Parti (2022) recently found that the frequency of social media use was a predictor of online fraud victimization for younger people instead of older ones and time spent online did not predict online victimization. According to the 49th statistical report on internet development in China (China Internet Network Information Center, 2022), as of December 2021, the average Chinese netizen spent 28.5 h online per week. The proportion of Chinese netizens using mobile phones to access the internet reached 99.7 percent, and mobile phones are the most important device for accessing the internet in China (China Internet Network Information Center, 2022). As a result, smartphone usage may be an indicator of online lifestyle to examine its predictive effect on online fraud victimization.

With respect to victims’ extrinsic features, the effects of negative life experiences and social support are usually examined. Negative life events can affect individual’s cognitive judgment, information processing, and decision-making ability. Fraudsters rely on cognitive biases or errors brought by negative life events to their victims to execute attacks and produce automatic emotional responses (Emami et al., 2019). Although a few studies have suggested a positive prediction effect of negative life experiences (Anderson, 2019; Emami et al., 2019), Sur et al. (2021) found that experiencing negative life events was not associated with the risk of self-reported fraud victimization, which makes the role of the factor ambiguous. Social support is the support and help that individuals can get through social interaction to reduce psychological stress response and improve social adaptability. The lack of social support will reduce the individual’s access to information resources and knowledge reserves, thus increasing the possibility of being deceived (Zhang et al., 2017). There are also inconsistent results about the effect of social support varying from negative, insignificant, to positive relationships (Beach et al., 2018; Fan and Yu, 2021). For example, James et al. (2014) argued for a negative association between susceptibility to scams and social support, while in Parti’s (2022) study living alone did not predict scam victimization and asking for help associated with a higher risk of being victims, which might suggest the null or even negative effect of social support. Sur et al. (2021) also revealed that higher consistent social support increases the average probability of fraud victimization.

Taken together, the findings above suggest that the victim’s inherent factors, such as demographic characteristics, psychological characteristics and lifestyle, as well as extrinsic factors, such as negative life experiences and social support, will play an important role in victimity and further predict fraud victimization. However, the findings of existing research were inconsistent has been mentioned above. To this end, the current study aimed to clarify the role of the above victimity features on victimization of online fraud in China. Based on the existing research, we hypothesized that there are significant differences between victims and non-victims in terms of demographic characteristics, psychological characteristics, lifestyle, negative life experiences and social support, and these factors can significantly predict online fraud victimization.

## 2 Materials and methods

### 2.1 Participants

The sample consisted of victims and non-victims of online fraud using a convenient sampling method in Zhejiang Province. The victim sample came from victims who reported the case to the police and the case needed to meet the following two criteria: (1) the victim received the fraud information online, and (2) the victim had transferred money to the scammer. The non-victim sample came from those who went to the police station to handle personal matters or report crimes other than fraud. The victims and non-victims were asked to fill out an online questionnaire. All the returned questionnaires were completed anonymously and voluntarily by the participants after their approval. We requested that participants under 18 years old fill out the questionnaires under the written approval and supervision of a guardian. In total, 1,027 valid questionnaires were collected. The participants included 512 males, accounting for 49.9%, and 515 females, accounting for 50.1%. The youngest was 12 years old, and the oldest was 80, for an average age of 35.74 (SD = 11.83). Among them, 504 people have experienced online fraud, accounting for 49.1%, and 523 people have not experienced online fraud victimization, accounting for 50.9%. The study procedures were approved by the Institutional Review Board of Zhejiang Police College.

### 2.2 Measures

#### 2.2.1 Basic information

Basic information consisted of demographic variables of the participants, which included age, gender and education, and whether the participants had victimization experience of online fraud (“No” = 0, “Yes” = 1).

#### 2.2.2 Impulsiveness

The degree of impulsiveness of participants was measured by the Barratt Impulse Scale (BIS-11) revised by Zhou et al. (2006). From the original scale, we selected nine items that are highly related to the degree of impulsiveness in acting and planning, such as “I act on impulse.” The scale adopts a 4-point scoring method from 1 (never) to 4 (always). The sum score ranges from 9 to 36. The higher the score is, the higher the impulsiveness of acting and planning. The fitting result of confirmatory factor analysis of this scale is χ^2^/*df* = 6.32, root-mean-square error of approximation (RMSEA) = 0.07, comparative fit index (CFI) = 0.94, and tucker-lewis index (TLI) = 0.91. In this study, the Cronbach’s alpha of the scale was 0.64.

#### 2.2.3 Trust tendency

Using the questionnaire of the deception tendency of the elderly compiled by Zhang et al. (2017), this study measured the trust tendency of the participants. Because of the wide age range of the research sample in this study, the items in the original questionnaire were deleted based on the previous interviews. The final questionnaire consists of 10 items, such as “How likely are you to ask the telecommunications bureau to help you when it calls to remind you of your arrears and is willing to help you to pay?” The questionnaire uses the 4-point scoring method, with 1 meaning very unlikely and 4 meaning very likely. The sum score ranges from 10 to 40. The higher the score is, the higher the trust tendency. The fitting result of confirmatory factor analysis of this scale is χ^2^/*df* = 4.81, RMSEA = 0.06, CFI = 0.96, and TLI = 0.93. In this study, the Cronbach’s alpha of the scale was 0.77.

#### 2.2.4 Smartphone usage

The smartphone dependence scale was used to measure the mobile phone usage of the participants (Kwon et al., 2013). Three items of the scale, such as “I frequently check my mobile phone to avoid missing conversations with others on social software (such as QQ or WeChat),” were selected based on the previous interviews. The scale adopts a 5-point scoring method, where 1 means totally disagree and 5 means totally agree. The sum score ranges from 3 to 15. The higher the score, the greater the smartphone usage. The fitting result of confirmatory factor analysis of this scale is χ^2^/*df* = 0.00, RMSEA = 0.00, CFI = 1.00, and TLI = 1.00. In this study, the Cronbach’s alpha of the scale was 0.67.

#### 2.2.5 Negative life experiences

The life events scale was used to measure the effects of total important life events in the past 6 months (Li et al., 2009). The scale includes 12 items, such as “I was hospitalized due to an accidental injury.” (See Supplementary Appendix A) the scale adopts a 6-point scoring method, with 0 representing no occurrence, 1 representing occurrence but no significant distress, 2 representing mild distress, 3 representing moderate distress, 4 representing somewhat severe distress, and 5 representing severe distress. The sum score ranges from 0 to 60. The higher the scale score, the more negative life events experienced in the past 6 months, and the higher the degree of distress. The fitting result of confirmatory factor analysis of this scale is χ^2^/*df* = 5.12, RMSEA = 0.06, CFI = 0.94, and TLI = 0.91. In this study, the Cronbach’s alpha of the scale was 0.82.

#### 2.2.6 Social support

The Social Support Revalued Scale revised by Xiao (1994) was used to measure the degree of social support (Xiao, 1994). Considering that some of the participants were unemployed, two items involving colleagues and organizations were deleted, so there were eight items on the scale, including three dimensions named subjective support, objective support, and support utilization. (See Supplementary Appendix B) the total score of social support is the sum of three dimensions. The sum score ranges from 10 to 58. The higher the score, the higher the level of social support. The fitting result of confirmatory factor analysis of this scale is χ^2^/*df* = 5.90, RMSEA = 0.07, CFI = 0.94, and TLI = 0.92. In this study, the Cronbach’s alpha of the scale was 0.81.

### 2.3 Statistical analysis

First, descriptive statistics were reported on demographic characteristics, impulsiveness, trust tendency, smartphone usage, negative life experiences, and social support. Second, the chi-square test and independent sample *t*-test were performed to examine the difference in demographic characteristics between victims and non-victims. Third, an independent sample *t*-test was conducted to compare the differences in social-psychological variables between the two groups. Finally, binary logistic regression analyses were performed to investigate the predictive effects of all the variables on online fraud victimization. The data were statistically analyzed using statistical product service solutions (SPSS) 24.0. The level of significant difference was *p* < 0.05.

## 3 Results

### 3.1 Preliminary analysis

Table 1 shows the demographic characteristics of the sample. All participants were divided into victims and non-victims according to their victimization experience of online fraud. For non-victims, the average age was 39.46 (SD = 11.73), while for victims, it was 31.89 (SD = 10.65). The proportion of males and females was similar for both groups (50.7% men for non-victims and 49.0% men for victims). A total of 16.3 and 54.9% of non-victims had junior high school education or less and junior college education or more, respectively, while for victims, the proportions were 31.9 and 42.3%, respectively.

**TABLE 1:** The demographic characteristics of non-victims and victims (*N* = 1,027).

Personal basic characteristics	Victimization		*t* or χ^2^
	**Non-victims** ***M* (SD) or *n* (%)**	**Victims** ***M* (SD) or *n* (%)**	
Age	39.46 (11.73)	31.89 (10.65)	10.83***
**Gender**			−0.53
Male	265 (50.7)	247 (49.0)	
Female	258 (49.3)	257 (51.0)	
**Education**			−6.31***
Junior high school or less	85 (16.3)	161 (31.9)	
Senior high school/ vocational high school/ technical secondary school	151 (28.8)	130 (25.8)	
Junior college/ Undergraduate	259 (49.5)	208 (41.3)	
Graduate or more	28 (5.4)	5 (1.0)	

***p < 0.001.

The independent sample *t*-test was conducted for age, and the chi-square test was conducted for gender and education to compare non-victims and victims. As shown in Table 1, the two groups had significant differences in age and education. Specifically, the victims were younger and less educated than the non-victims, supporting our hypothesis. However, there was no significant difference between the two groups based on gender, which is inconsistent with the hypothesis.

### 3.2 Differences between victims and non-victims in social-psychological variables

Table 2 shows the mean scores and standard deviations of the social-psychological variables, as well as the difference between victims and non-victims conducted by independent sample *t*-test. The results showed significant differences between the two groups in impulsiveness, trust tendency, smartphone usage, negative life experiences, and social support. Victims had significantly greater impulsiveness, trust tendency, and smartphone usage frequency than non-victims, as well as experiencing significantly more negative life events and having less social support, which is consistent with our hypothesis.

**TABLE 2:** Differences between victims and non-victims in social-psychological variables (*N* = 1,027).

Variable		*M* (SD)	*t*	*p*
Impulsiveness	Non-victims	19.34 (3.86)	−8.12	<0.001
	Victims	21.17 (3.35)		
Trust tendency	Non-victims	18.01 (4.39)	−6.93	<0.001
	Victims	20.02 (4.88)		
Smartphone usage	Non-victims	9.82 (3.47)	−2.02	0.044
	Victims	10.27 (3.66)		
Negative life experiences	Non-victims	6.13 (6.43)	−5.51	<0.001
	Victims	8.73 (8.48)		
Social support	Non-victims	35.71 (7.52)	10.79	<0.001
	Victims	30.54 (7.83)		

### 3.3 Logistic regression analysis of the measured variables

**FIGURE 1:**
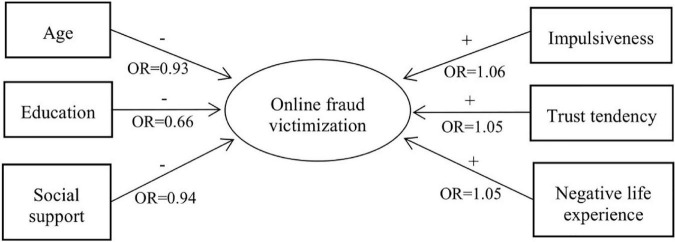
The predictive effects of the measured variables on online fraud victimization. OR, odds ratio.

To analyze the predictive effects of demographic and social-psychological characteristics on online fraud victimization, binary logistic regression was conducted with victimization as the dependent variable; age, gender, education, impulsiveness, trust tendency, smartphone usage, negative life events experience, and social support as the independent variables; and non-victims as the reference category. As shown in Table 3, impulsiveness, trust tendency, and negative life events experience have significant positive predictive effects on online fraud victimization, while age, education, and social support have significant negative predictive effects on victimization. Gender and smartphone usage as predictors yielded no significant effects; therefore, a second regression analysis was performed with victimization as the dependent variable; age, education, impulsiveness, trust tendency, negative life events experience, and social support as the independent variables; and non-victims as the reference category. The results are shown in Table 4. Specifically, younger and less educated people had a significantly higher likelihood of being victims of online fraud. Impulsiveness, trust tendency and negative life events experience are significantly more likely to lead to an individual being victimized by online fraud. Social support is negatively correlated with victimization. The results shown in Figure 1 indicate that the younger the age is, the lower the education level, the stronger the impulsiveness, the higher the level of trust tendency, the greater the influence of negative life events, the lower the level of social support, and the more likely one is to become a victim of online fraud.

**TABLE 3:** Factors predicting online fraud victimization in the first binary logistic regression analysis (*N* = 1,027).

Factors	*B*	Exp (*B*) (95% CI)
Age	−0.07***	0.93 (0.92–0.95)
Gender	0.13	1.14 (0.85–1.52)
Education	−0.42***	0.66 (0.60–0.73)
Impulsiveness	0.06*	1.06 (1.01–1.11)
Trust tendency	0.05**	1.05 (1.02–1.09)
Smartphone usage	0.00	1.00 (0.96–1.05)
Negative life experiences	0.05***	1.05 (1.03–1.07)
Social support	−0.06***	0.94 (0.93–0.96)

*p < 0.05, **p < 0.01, ***p < 0.001.

**TABLE 4:** Factors predicting online fraud victimization in the second binary logistic regression analysis (*N* = 1,027).

Factors	*B*	Exp (*B*) (95% CI)
Age	−0.07***	0.93 (0.92–0.95)
Education	−0.42***	0.66 (0.59–0.73)
Impulsiveness	0.06**	1.06 (1.02–1.11)
Trust tendency	0.05**	1.05 (1.02–1.09)
Negative life experiences	0.05***	1.05 (1.03–1.07)
Social support	−0.06***	0.94 (0.93–0.96)

**p < 0.01, ***p < 0.001.

## 4 Discussion

Online fraud is currently the main form of crime against property in China. The effectiveness of victimization prevention largely depends on the investigation of factors contributing to the victimity of the victims. The present study investigated the association of demographic and social-psychological factors with online fraud victimization to examine the role of these inherent and extrinsic factors in the victimity of fraud victims. The univariate analyses found that there was a significant difference in age, education, impulsiveness, trust tendency, smartphone usage, negative life experiences, and social support between victims and non-victims. This was mostly consistent with our hypotheses, except that gender exhibited a null effect. With the logistic regression analysis, age, education, impulsiveness, trust tendency, negative life experiences, and social support were found to be significant predictive factors of victimization.

### 4.1 Inherent factors of victimity associated with victimization

The independent sample *t*-test shows that victims are significantly younger and less educated than non-victims. The logistic regression also suggests a negative predictive role of the two demographic characteristics. The results support our hypothesis that younger age and less education are associated with a higher risk of fraud victimization. According to the 48th statistical report on internet development in China (China Internet Network Information Center, 2021), Chinese netizens aged 30–39 accounted for 20.3%, the highest proportion among all ages, followed by those aged 40–49 (18.7%) and 20–29 (17.4%). The younger age of the victims may correlate with the greater usage of the internet in young people, which makes them more easily exposed to fraud messages. Education may be related to knowledge of the internet and internet scams. Wright and Marett (2010) suggested that people with higher levels of computer self-efficacy, web experience, and security knowledge were less susceptible to phishing attempts. Considering that overconfidence of high education people in not being defrauded may be related to high risk of victimization (Parti, 2022), it is important to make sure that people have the knowledge to identify scams and handle large amounts of money. Our results do not support the hypothesis that gender is a predictor of victimization. Although the present study shows no gender difference between victims and non-victims, the effect may be displayed when discussed by the type of fraud. For example, Whitty (2020) found that women were much more likely to be victims of consumer scams, while men were more likely to be victims of investment scams. This may be due to the different social roles and needs of men and women that leads to the gender gap in the victimity of the specific type of online fraud. Future research may explore the demographic characteristics associated with different types of online fraud in China to more deeply understand victimity and more precisely prevent the crime.

For psychological characteristics associated with fraud victimization, our results show a positive predictive role of impulsiveness and trust tendency, supporting our hypothesis that greater impulsiveness and trust tendency are associated with a higher risk of fraud victimization. Impulsiveness is often regarded as a tendency to respond quickly to unplanned internal and external stimuli, which often causes adverse effects due to a lack of consideration of behavioral consequences (An and Jiang, 2020). When individuals are impulsive, their decision-making is more susceptible to the influence of others rather than their own rational thinking, and they are more likely to trust the defrauders under elaborate scams. However, individuals with low levels of impulsiveness have a better ability to identify potential fraudulent information (Norris et al., 2019). Some studies have examined the relationship between internet or mobile phone dependence and impulsiveness (Cao et al., 2007; Billieux et al., 2010; An and Jiang, 2020). The results showed a positive correlation, which may explain why impulsive people are more likely to be victims of online fraud. Trust tendency, also known as the general level of trust, refers to the inherent trust level of an individual without any known information about others (Dinesen, 2012). Our results support a previous study showing that victims of online fraud often have a higher level of trust in others (Zhao et al., 2020). The study of network communication situations also showed that the higher the level of trust tendency, the easier it is trust other individuals and other groups (Teo and Liu, 2007). Compared to the participants in the study of Carter and Weber (2010), who found that high trusters were significantly better than low trusters at detecting lies through watching videos of job interviews, the victims of communication and internet fraud have fewer clues for judgment, thus making people with a high trust tendency more likely to be deceived.

o investigate the association between lifestyle and victimization, the current study examines the role of smartphone usage on fraud victimization. The results show that although smartphone usage is not a significant predictor of online fraud, it is positively correlated with victimization. According to lifestyle exposure theory, people will have different chances of encountering crime risks based on different lifestyles. If some lifestyles have more contact opportunities with potential crimes, or they are often in a situation where crimes occur, their risk of being victimized will be higher (Li, 2010). Further, once people provide money to scammers, they may be in a higher risk of repeat victimization (Balleisen, 2018; Parti, 2022). Individuals’ dependence on smartphones actually reflects their lifestyle of frequent occupational activities and entertainment activities through the internet, which leads to an increased risk of exposure to online fraud; thus, they are more likely to become the target of defrauders. In addition, Holtfreter et al. (2008) found that the differences in personal basic characteristics such as age, gender, and socioeconomic status may actually reflect the differences in daily activities such as consumption behavior, thus affecting their victimization. On the other hand, online consumption behavior increases the risk of being targeted for fraud (Holtfreter et al., 2008). In the present study, the null prediction of smartphone usage may be due to the lack of typicality in the question setting. Parti (2022) measured the frequency of social media use by the variable named online services and found that using dating services and playing online games made younger people vulnerable to online fraud. Future research can use more specific questions to investigate the role of lifestyle especially online activities in fraud victimization.

The inherent factors of victimity associated with victimization indicates that online fraud victimization is largely related to the victim’s own stable internal characteristics. In some cases, it is not because the victim’s initiative leads to the victimization, but their characteristics make them more vulnerable to the crime, which may also partly explain the repeat victimization. The results suggest that relevant departments should pay special attention to the population with the above characteristics, and carry out targeted education to prevent them from being deceived as much as possible.

### 4.2 Extrinsic factors of victimity associated with victimization

Negative life events are considered to be a risk factor for fraud victimization in some studies (Deliema, 2015; Anderson, 2019; Emami et al., 2019). The current study finds that negative life events positively predict victimization, supporting our hypothesis. There are several explanations for this result. First, negative life events may increase fraud vulnerability by reducing social support and the number of social activities out of the home so that people may spend more time on the internet (Sur et al., 2021). Second, people who experience negative life events such as divorce or unemployment are more likely to have a need to make friends, get a job or get a loan, increasing their exposure to scams. Emami et al. (2019) found that victims of online fraud were more likely than non-victims to experience the breakdown of marriage or other intimate relationships. The third explanation is that according to the Elaboration Likelihood Model (ELM; Petty and Cacioppo, 1986), individual differences such as mood at the time of receiving a message are heavily correlated with the depth of processing that a person engages in when encountering a potential scam message (Norris and Brookes, 2020). The negative state relief model (NSR; Cialdini et al., 1973) implies that individuals in a negative mood state are more likely to respond to positively framed scam messages to relieve their negative mood (Norris and Brookes, 2020). The role of negative life events in our study may be realized through the effect of negative mood caused by the events on scam message processing. Considering the view that the influence of emotional factors can occur through different primary mechanisms and in different contexts (Norris and Brookes, 2020), future research should investigate the role of different negative life events in different types of online fraud.

The present study shows a significant correlation between social support and fraud victimization. Specifically, people with less social support have a higher risk of fraud victimization. Social support refers to the spiritual or material support and help received by individuals from society, including the support provided by relatives and friends, colleagues and neighbors, or public welfare organizations and enterprise associations (He, 2019). If victims of online fraud receive less support from the real society, they are more likely to blindly pursue the scope of social activities online, thus connecting with strangers online. The possibility of being exposed to the crime situation increases accordingly. In addition, less social support will leave victims without effective supervisors. According to routine activity theory, a crime occurs in the convergence of a motivated offender, a suitable target, and absent or ineffective prevention efforts (Cohen and Felson, 1979; Cohen et al., 1981). If a victim talks about the incident with someone else before or during the victimization, that person may act as a “protector” who effectively prevents the occurrence and development of fraud. For individuals who lack social support, this is a key deficiency, thus promoting the successful realization of the crime. For the result in the study of Sur et al. (2021) that perceived that social support was positively associated with victimization of old people, it may be that social support increased the odds of remembering and reporting fraud instead of the risk of being victimized. Since reporting or asking for help does little to prevent scam victimization (Parti, 2022), the specific forms of social support need to be further explored.

The role of extrinsic factors of victimity on victimization suggest that social connection plays an important role in preventing online fraud. Enhancing social connection can improve the individuals’ social support level, and reduce the online social demands and information processing deviation caused by negative emotions when individuals experience negative life events, so as to effectively prevent victimization.

## 5 Limitations

This study investigated the correlation between inherent and extrinsic factors of victimity, including age, gender, education, impulsiveness, trust tendency, lifestyle, negative life events and social support, and online fraud. The results show significant relations for all of the factors except for gender and lifestyle. As mentioned above, the null effect may be due to the lack of research on the influence of these factors on different types of fraud. Since there are many different methods of committing fraud in China and different people have different needs and characteristics, it may be that different types of people fall for different types of scams (Whitty, 2020). The analysis could have benefited from investigating the risk factors for victimization according to different types of fraud. The lack of sample representativity and generalizability caused by the sampling method in this study is also one of the limitations. Future research need to adopt more appropriate sampling methods and further expand the sample size. In addition, the current study is also limited by the self-report measure, which may be affected by the participants’ interpretation of questions, the perception of their own behaviors, social expectations, and so on (De Leeuw et al., 1999; Deevy and Beals, 2013; Judges et al., 2017; Williams et al., 2017). Besides, there may be a small part of non-victims who do not know that they have been deceived, so they are misclassified in the questionnaire which may affect the results. Future research could consider experimental or behavioral methods to investigate the risk factors for fraud victimization and their effect mechanisms to obtain more objective data.

## 6 Conclusion and implications

Considering the current severe situation and prevention bottlenecks of online fraud in China, this study investigated the risk factors correlated with fraud victimization from the perspective of victimity. The results show that younger age, less education, greater impulsiveness, greater trust tendency, more negative life experiences, and less social support are associated with a higher risk of online fraud victimization. The present findings provide a further understanding of the victimity of online fraud victims and demonstrate a social-psychological profile of the victims. In terms of practice, some preventative measures can be put forward based on these findings. People who meet the profile of online fraud victims should be screened and educated for fraud prevention. Since social support plays an important role in both the causes and the consequences of criminal behavior (Cullen, 1994), a social support system could be established to provide effective support for online fraud victims and potential victims to reduce the risk of victimization and repeat victimization of online fraud.

## Data availability statement

The raw data supporting the conclusions of this article will be made available by the authors, without undue reservation.

## Ethics statement

The studies involving human participants were reviewed and approved by Institutional Review Board of Zhejiang Police College. Written informed consent to participate in this study was provided by the participants’ legal guardian/next of kin. Written informed consent was obtained from the individual(s), and minor(s)’ legal guardian/next of kin, for the publication of any potentially identifiable images or data included in this article.

## Author contributions

ZZ designed the study. ZZ and ZY collected and analyzed the data and wrote the manuscript. Both authors contributed to the article and approved the submitted version.
