# Supplementary material for: The role of social-psychological factors of victimity on victimization of online fraud in China
Source: Front Psychol. 2022 Dec 23;13:1030670. doi: 10.3389/fpsyg.2022.1030670 (PMC9822720; doi:10.3389/fpsyg.2022.1030670)
Supplement: Supplementary file 1 [file Presentation_1.pdf]

Appendix A. Life events scale

Have your living circumstances changed over the past six months? Please tick off the appropriate numbers of each item.

Over the past six months	Did not happen	Happened, and the degree of distress				
		None	Mild	Moderate	Somewhat severe	Severe
Been in a car accident	0	1	2	3	4	5
Been hurt in another kind of accident or sick in the hospital	0	1	2	3	4	5
Friend very sick, hurt, or died	0	1	2	3	4	5
Seen someone else get hurt	0	1	2	3	4	5
Parents broke things or hurt each other	0	1	2	3	4	5
Separated or divorced from my partner, or parents separated or divorced	0	1	2	3	4	5
Someone in the family in the hospital (hurt or sick)	0	1	2	3	4	5
Someone in the family died	0	1	2	3	4	5
Someone in the family went out to work	0	1	2	3	4	5
Someone in the family got married	0	1	2	3	4	5
Moved to a new place	0	1	2	3	4	5
Changed a new job	0	1	2	3	4	5

Appendix B. Social Support Revalued Scale

The following questions are intended to reflect the support you have received from the society. Please select the appropriate options according to the specific requirements of each question and your actual situation.

1.How many close friends do you have that you can count on for support? (Just choose one)

○None ○1~2 ○3~5 ○6 or more

2.What's your living condition over the past year? (Just choose one)

○Living alone ○Living with strangers most of time

○Living with schoolmate, colleague, or friend ○Living with family

3.What's the relationship between you and your neighbor? (Just choose one)

○We don't care about each other ○A little care when running into difficulties

○Some neighbors care about me ○Most of the neighbors care about me

4.Support and care from family members: (tick off in the appropriate box)

	None	Very few	Moderate	A lot
Spouse (lover)				
Parents				
Children				
Brothers and sisters				
Other members				

5.In the past, the sources of financial support or problem solving help you have received in an emergency or difficult situation include:

○None

○The following sources (you can choose more than one)： ○Spouse ○Other family members ○friends ○Relatives ○Colleagues ○Work unit ○Official or semi-official organizations such as caucus unions ○Religious, social and other non-official organizations ○Others___________

6.In the past, the sources of comfort and concern you have received in an emergency or difficult situation include:

○None

○The following sources (you can choose more than one)： ○Spouse ○Other family members ○friends ○Relatives ○Colleagues ○Work unit ○Official or semi-official organizations such as caucus unions ○Religious, social and other non-official organizations ○Others___________

7.What's the way you talk about your troubles? (Just choose one)

○Never confide in anyone ○Only confide in 1-2 closest friends

○Talk about the troubles if my friends ask ○Take the initiative to talk about my troubles for understanding and support

8.What's the way you ask for help when you are in trouble? (Just choose one)

○Only on my own, do not accept help from others ○Rarely ask for help

○Ask for help sometimes ○Always ask for help
